# An L-shaped link between the composite dietary antioxidant index and human papillomavirus infection in women: a US population-based study

**DOI:** 10.3389/fnut.2025.1604908

**Published:** 2025-09-10

**Authors:** Yuhua Li, Yulin Zheng, Jinhua Zhao, Yuanyuan Cao, Xiaofei Meng, Xiaoyan Liu, Xiaolan Wang, Lili Zhang

**Affiliations:** ^1^Department of Infectious Diseases, Beijing You’an Hospital, Capital Medical University, Beijing, China; ^2^Department of Critical Medicine, Beijing You’an Hospital, Capital Medical University, Beijing, China; ^3^Department of Nursing, Beijing You’an Hospital, Capital Medical University, Beijing, China

**Keywords:** human papillomavirus infection, composite dietary antioxidant index, dietary antioxidants, dose–response, NHANES

## Abstract

**Objective:**

This study intended to inspect the link between a comprehensive dietary antioxidant index (CDAI) and human papillomavirus (HPV) infection in US women.

**Methods:**

The link between CDAI and HPV infection was analyzed by weighted univariate and multivariate regression models, restricted cubic spline (RCS), and subgroup analyzes using the NHANES data from 2003 to 2016.

**Results:**

Data from 8,115 subjects were included, with a weighted prevalence of HPV infection of 38.01%. After adjusting for all covariates, HPV infection decreased by 2% for each 1-unit rise in CDAI [(95%CI: 0.96, 0.99), *p* = 0.042]. RCS results elicited a non-linear link (P-non-linear = 0.043). VE and zinc intake were negatively linked to HPV infection (both *p* < 0.05). Subgroup analysis noted a notable interaction of marital status in the link between CDAI and HPV infection (P for interaction = 0.011).

**Conclusion:**

CDAI is negatively linked to HPV infection. American women can enhance their intake of antioxidant-rich foods, especially those rich in zinc and vitamin E, to reduce HPV infection risk and enhance antioxidant defenses.

## 1 Introduction

Human Papillomavirus (HPV) is a prevalent sexually transmitted virus. Studies have evinced that the average lifetime probability of HPV infection is 84.6% for women and 91.3% for men (1). Although most HPV infections are self-limiting and usually clear on their own within a short period, a subset of individuals experience persistent infections, which greatly enhances carcinogenesis risk (2). Studies (3–5) have demonstrated that specific types of HPV infections are key in the development of cervical cancer in women and other cancers, including vulvar., anal, oral, and head and neck cancers. Effective prevention methods based on vaccination and screening are fundamentally changing HPV-related cancer prevention and control. However, effective HPV vaccination and screening still face significant public health challenges globally (6). Thus, it is particularly important to explore other feasible prevention and control strategies.

Redox balance is critical for maintaining cellular homeostasis and immune function. When the production of reactive oxygen species (ROS) and reactive nitrogen species (RNS) exceeds the scavenging capacity of the antioxidant system, a redox imbalance occurs. When this imbalance tilts toward oxidation, it triggers oxidative stress, leading to oxidative damage to lipids, proteins, and DNA. Subsequently, it induces chronic inflammatory responses and suppresses the body’s antiviral immunity, thereby creating conditions for persistent HPV infection, viral integration, and carcinogenesis (7). Research findings have demonstrated that multiple high-risk HPV (hrHPV) early proteins (such as E1, E2, E6) can promote excessive ROS/RNS production by reducing the levels of certain antioxidant enzymes (such as superoxide dismutase, catalase, or glutathione peroxidase) and glutathione, exacerbating redox imbalance and DNA damage, thereby promoting the carcinogenic process of the virus (8). Dietary antioxidant-rich components, like vitamin C (VC), vitamin E (VE), carotenoids, and plant polyphenols, can enhance immune function, reduce oxidative stress, and improve cellular DNA repair (9). Thereby, the potential benefit of dietary antioxidants in the prevention and clearance of HPV infection has recently attracted much attention (9).

Currently, most studies have focused on the link between a single antioxidant component and HPV infection (10, 11). However, the overall effect of dietary antioxidants may not be simply added, and there may be potential synergistic or antagonistic effects between different antioxidants (12). The composite dietary antioxidant index (CDAI) of various dietary antioxidants, like vitamins A (VA), VC, and VE, selenium, zinc, and carotenoids, can be viewed as a measure of total dietary intake of antioxidants (13). It has been extensively used in the study of the risk of various diseases (14, 15). In 2020, Barchitta et al. (16) first applied CDAI to assess the overall effect of dietary antioxidants on HPV infection. The study included 241 Italian women and showed an 8% reduction in hrHPV infection risk for each 1-unit rise in CDAI, after adjusting for relevant confounders. However, dietary structure, lifestyle, and ethnic differences may substantially influence the link between dietary antioxidants and HPV infection risk. Therefore, investigating the potential mechanism of CDAI and HPV infection in large-sample studies across different populations remains an important direction for future research.

This paper relied on the NHANES database in women aged 20–59 years to ascertain the link between CDAI and HPV infection risk.

## 2 Methods

### 2.1 Study populations and design

The data were sourced from 2003 to 2016 NHANES, a publicly available database for the data collection process, analysis guidelines, and complete dataset. The NHANES project was ratified by the Research Ethics Review Board of the National Center for Health Statistics and rigorously evaluated. All subjects signed a written informed consent form confirming voluntary participation. As the NHANES data were deidentified and anonymized, no additional ethical approvals or supplemental informed consent were required for subsequent analyzes of this study. The relevant ethical review approval documents are presented on the official NHANES website.

**Figure 1:**
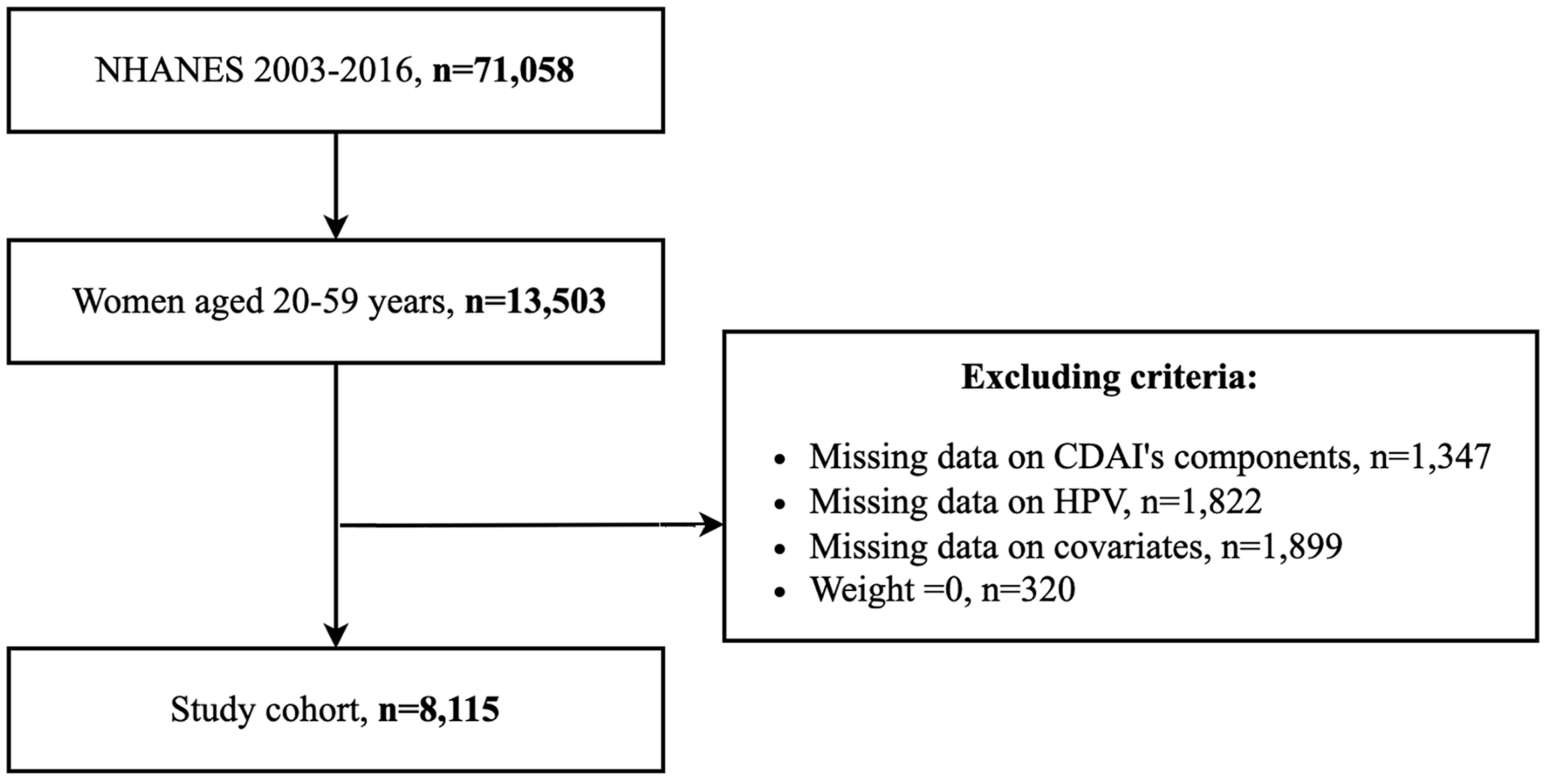
Study flowchart.

The study population consisted of women aged 20–59 years. Individuals with missing CDAI calculations, HPV testing records, and covariates, and individuals with a weight of 0 were excluded. 8,115 eligible participants were finally enrolled, and the inclusion process is displayed in Figure 1.

### 2.2 Variables

#### 2.2.1 CDAI

The CDAI was reckoned by averaging the VA, VC, VE, zinc, selenium, and carotenoids derived from the two interviews, then standardizing the means, and finally summing them (17). Carotenoids cover *α*-carotene, *β*-carotene, *β*-cryptoxanthin, lutein, lycopene, and zeaxanthin (18). They were categorized into three groups using triplet quantities, denoted by T1, T2, and T3.

#### 2.2.2 HPV measurement

A positive test result for any of the 37 types of HPV (6, 11, 16, 18, 26, 31, 33, 35, 39, 40, 42, 45, 51–56, 58, 59, 61, 62, 64, 66–73, 81, 82, 83, 84, 89, and IS39) was considered HPV infection in the study sample (19). Additionally, to further refine the classification, if the result for any hrHPV type (including HPV 16, 18, 31, 33, 35, 39, 45, 51, 52, 56, 58, and 59) was positive, it was classified as hrHPV infection; conversely, if the result was positive for other HPV genotypes not listed above, it was classified as low-risk HPV (lrHPV) infection.

#### 2.2.3 Covariates

Demographic variables included age, sex, race, poverty-to-income ratio (PIR), education level, marital status, and BMI. The definition of diabetes mellitus (DM) was based on one of the following indicators: glycosylated hemoglobin ≥6.5%; fasting glucose ≥126 mg/dL; or answered yes to one of the questions “Are you using insulin?,” “Are you diagnosed with DM?” or “Are you taking hypoglycemic medication?” (20). Smoking status was categorized into nonsmokers (less than 100 cigarettes in their lifetime), quitters (smoked over 100 cigarettes but do not currently smoke), and current smokers (smoked over 100 cigarettes and are still smoking) (21). Consumption of at least 12 alcoholic beverages per year was considered alcohol consumption (10). Variables such as whether the study population had taken oral contraceptives, age at first sex, and male sexual partners in the last year were also collected.

### 2.3 Statistical analyzes

Statistical analysis was made in R 4.3.3. The Kolmogorov–Smirnov normality test was employed to check the distribution of continuous variables. For continuous variables in normal distribution, they were delineated as mean ± standard deviation and processed via *t*-tests. For variables not in normal distribution, they were manifested as median and interquartile spacing [M (P25, P75)] and processed via the Mann–Whitney U test. Categorical variables were delineated as number (*n*) and percentage (%) and compared via the chi-square test (x^2^) or Fisher exact test.

The links of CDAI and its components with HPV infection risk were determined through logistic regression. Odds ratios (ORs) and their 95% confidence intervals (CIs) were reckoned. No factors were adjusted in Model 1. Model 2 was adjusted for age and race; and Model 3 further considered education level, BMI, marital status, PIR, alcohol consumption, smoking status, DM, oral contraceptives, age at first sex, and male sexual partners in the last year. In Model 3, the nonlinear association of CDAI with HPV infection was checked via the restricted cubic spline (RCS) method. Subgroup analyzes were implemented to determine heterogeneity and interactions in specific populations. Finally, to reduce the potential impact of HPV vaccination on the results, we further excluded all participants who reported receiving HPV vaccination and conducted a sensitivity analysis to assess the robustness of the results. A two-sided *p* < 0.05 inferred statistical significance.

## 3 Results

### 3.1 Population characteristics

8,115 participants were enrolled, and 3,337 of them had HPV infection, with a weighted prevalence of 38.01%. The median age was 40.0 years [Q1–Q3: 30.0–50.0]. 8.51% of the participants were Mexican American, 11.91% were non-Hispanic White, 65.59% were non-Hispanic White, 5.21 were other Hispanic, and 6.78% were other races (Table 1). Compared to those without HPV infection, those with HPV infection were likely to be younger (42.00 [IQR: 31.00–51.00] vs. 38.00 [IQR: 27.00–48.00], *p* < 0.001), have lower education level (*p* = 0.004), more likely to consume alcohol (*p* < 0.001), current smokers (*p* < 0.001), DM (*p* = 0.018), lower CDAI (0.00 [IQR: −2.24–2.37] vs. − 0.87 [IQR: −2.94–1.80], *p* < 0.001), and lower intake of VA, VC, VE, zinc, and carbohydrates (all *p* < 0.01). In addition, there were pronounced differences in race, marital status, PIR, oral contraceptives, age at first sex, and male sexual partners in the last year (all *p* < 0.05) (Table 1).

**Table 1:** Baseline characteristics of the study population according to HPV.

Characteristic	Overall	No HPV infection	HPV infection	*p*-value
Age, years	40.00 (30.00, 50.00)	42.00 (31.00, 51.00)	38.00 (27.00, 48.00)	<0.001
BMI, kg/m^2^				0.900
<25	2,629 (36.42%)	1,546 (36.27%)	1,083 (36.68%)	
25–30	2,172 (27.03%)	1,276 (27.30%)	896 (26.60%)	
≥30	3,314 (36.54%)	1,916 (36.44%)	1,398 (36.72%)	
Race				<0.001
Mexican American	1,338 (8.51%)	850 (8.78%)	488 (8.06%)	
Non-Hispanic Black	1,746 (11.91%)	746 (7.95%)	1,000 (18.37%)	
Non-Hispanic White	3,582 (67.59%)	2,244 (71.05%)	1,338 (61.94%)	
Other Hispanic	738 (5.21%)	409 (4.65%)	329 (6.13%)	
Other Race	711 (6.78%)	489 (7.57%)	222 (5.49%)	
Education level				0.004
<High school diploma	1,490 (12.58%)	828 (11.37%)	662 (14.56%)	
≥High school diploma	6,625 (87.42%)	3,910 (88.63%)	2,715 (85.44%)	
Marital status				<0.001
Married/cohabitation	4,946 (65.05%)	3,359 (74.45%)	1,587 (49.72%)	
Unmarried	1,708 (18.44%)	726 (13.32%)	982 (26.80%)	
Widow/divorce/separation	1,461 (16.51%)	653 (12.23%)	808 (23.48%)	
PIR				<0.001
<1.3	2,561 (22.15%)	1,258 (17.56%)	1,303 (29.64%)	
1.3–3	2,332 (26.30%)	1,343 (25.27%)	989 (27.98%)	
> = 3	3,222 (51.55%)	2,137 (57.17%)	1,085 (42.37%)	
Alcohol drinking				<0.001
Yes	5,505 (73.98%)	3,036 (71.03%)	2,469 (78.79%)	
No	2,610 (26.02%)	1,702 (28.97%)	908 (21.21%)	
Smoking status				<0.001
Current smoker	1,752 (22.22%)	758 (16.95%)	994 (30.82%)	
Former smoker	1,295 (17.80%)	788 (19.06%)	507 (15.75%)	
Never smoker	5,068 (59.97%)	3,192 (63.98%)	1,876 (53.44%)	
Diabetes mellitus				0.018
Yes	735 (7.51%)	447 (8.25%)	288 (6.30%)	
No	7,380 (92.49%)	4,291 (91.75%)	3,089 (93.70%)	
Have taken birth control pills				0.800
Yes	6,258 (81.50%)	3,637 (81.61%)	2,621 (81.32%)	
No	1,857 (18.5%)	1,101 (18.39%)	756 (18.68%)	
How old when first had sex, years				<0.001
<18	4,645 (57.40%)	2,395 (51.28%)	2,250 (67.37%)	
≥18	3,470 (42.60%)	2,343 (48.72%)	1,127 (32.63%)	
Male sex partners/year	1.00 (1.00, 1.00)	1.00 (1.00, 1.00)	1.00 (1.00, 1.00)	<0.001
CDAI	−0.33 (−2.50, 2.11)	0.00 (−2.24, 2.37)	−0.87 (−2.94, 1.80)	<0.001
VA	506.50 (317.50, 764.50)	535.00 (331.50, 795.00)	464.50 (284.50, 713.00)	<0.001
VC	59.35 (28.55, 107.35)	62.95 (31.35, 111.20)	53.05 (24.10, 99.65)	<0.001
VE	6.56 (4.57, 9.32)	6.75 (4.79, 9.59)	6.23 (4.31, 8.85)	<0.001
Se	93.50 (70.60, 120.35)	94.85 (71.50, 120.35)	90.90 (68.50, 120.50)	0.083
Zn	9.37 (6.99, 12.23)	9.65 (7.28, 12.37)	8.80 (6.60, 11.86)	<0.001
Carotenoids	6,698.00 (3,114.00, 12,225.50)	7,249.00 (3,513.00, 12,737.00)	5,821.50 (2,576.00, 11,478.00)	<0.001
Alpha_carotene	83.50 (23.00, 465.00)	102.50 (26.00, 535.00)	63.00 (18.00, 355.50)	<0.001
Beta_carotene	1,115.50 (426.50, 2,780.50)	1,285.00 (479.00, 2,964.00)	904.50 (360.50, 2,413.00)	<0.001
Beta_cryptoxanthin	36.50 (12.00, 96.50)	40.00 (13.50, 107.50)	32.50 (10.50, 81.50)	<0.001
Lycopene	2,624.50 (799.00, 6,432.00)	2,727.50 (832.50, 6,862.00)	2,400.00 (702.50, 5,978.50)	<0.001
Lutein_zeaxanthin	814.50 (421.00, 1,713.50)	871.00 (453.50, 1,844.50)	730.00 (372.50, 1,496.00)	0.010

HPV, human papillomavirus; BMI, body mass index; PIR, poverty-to-income ratio; CDAI, comprehensive dietary antioxidant index; VA, vitamin A; VC, vitamin C; VE, vitamin E; Se, selenium; Zn, zinc.

### 3.2 Link between CADI and HPV infection and RCS analysis

Model 3 delineated that for every 1-unit rise in CDAI, there was a subsequent 2.0% decrease in HPV infection risk [(95% CI: 0.96, 0.99), *p* = 0.042]. CDAI was further categorized by tertiles. In Models 1 and 2, the T2 and T3 groups were statistically different compared with the T1 group (both *p* < 0.05) and showed a decreasing trend (both P for trend <0.0001). In Model 3, only the T3 group was greatly different from the T1 group (*p* = 0.014), again showing a decreasing trend (P for trend = 0.042) (Table 2).

**Table 2:** The relationship between CDAI and HPV.

Participants	Model 1	*p*-value	Model 2	*p*-value	Model 3	*p*-value
	OR (95%CI)		OR (95%CI)		OR (95%CI)	
HPV vs. No HPV						
CDAI	0.96 (0.94, 0.98)	<0.001	0.96 (0.95, 0.98)	<0.001	0.98 (0.96, 0.99)	0.042
Tertiles						
T1	Ref		Ref		Ref	
T2	0.73 (0.62, 0.87)	<0.001	0.76 (0.64, 0.90)	0.002	0.89 (0.74, 1.06)	0.200
T3	0.65 (0.55, 0.77)	<0.001	0.69 (0.59, 0.82)	<0.001	0.80 (0.67, 0.95)	0.014
*p* for trend	<0.001		<0.001		0.042	
hrHPV vs. lrHPV						
CDAI	0.99 (0.96, 1.01)	0.297	0.99 (0.96, 1.01)	0.289	0.99 (0.96, 1.01)	0.303
Tertiles						
T1	Ref		Ref		Ref	
T2	1.04 (0.84, 1.29)	0.732	1.08 (0.87, 1.33)	0.494	1.07 (0.86, 1.34)	0.552
T3	0.91 (0.70, 1.17)	0.439	0.90 (0.69, 1.16)	0.399	0.90 (0.69, 1.17)	0.421
*p* for trend	0.530		0.345		0.384	

No factors were adjusted in Model 1. Model 2 was adjusted for age and race; and Model 3 further considered education level, BMI, marital status, PIR, alcohol consumption, smoking status, DM, oral contraceptives, age at first sex, and male sexual partners in last year. CDAI, comprehensive dietary antioxidant index; HPV, human papillomavirus; hrHPV, high-risk HPV; lrHPV, low-risk HPV; OR, odds ratio; CI, confidence interval.

Compared with lrHPV infection, CDAI was not significantly associated with hrHPV infection risk in any of the three models. Furthermore, when CDAI was classified into tertiles, there were no statistically significant differences between each tertile group and the reference group (Table 2).

**Figure 2:**
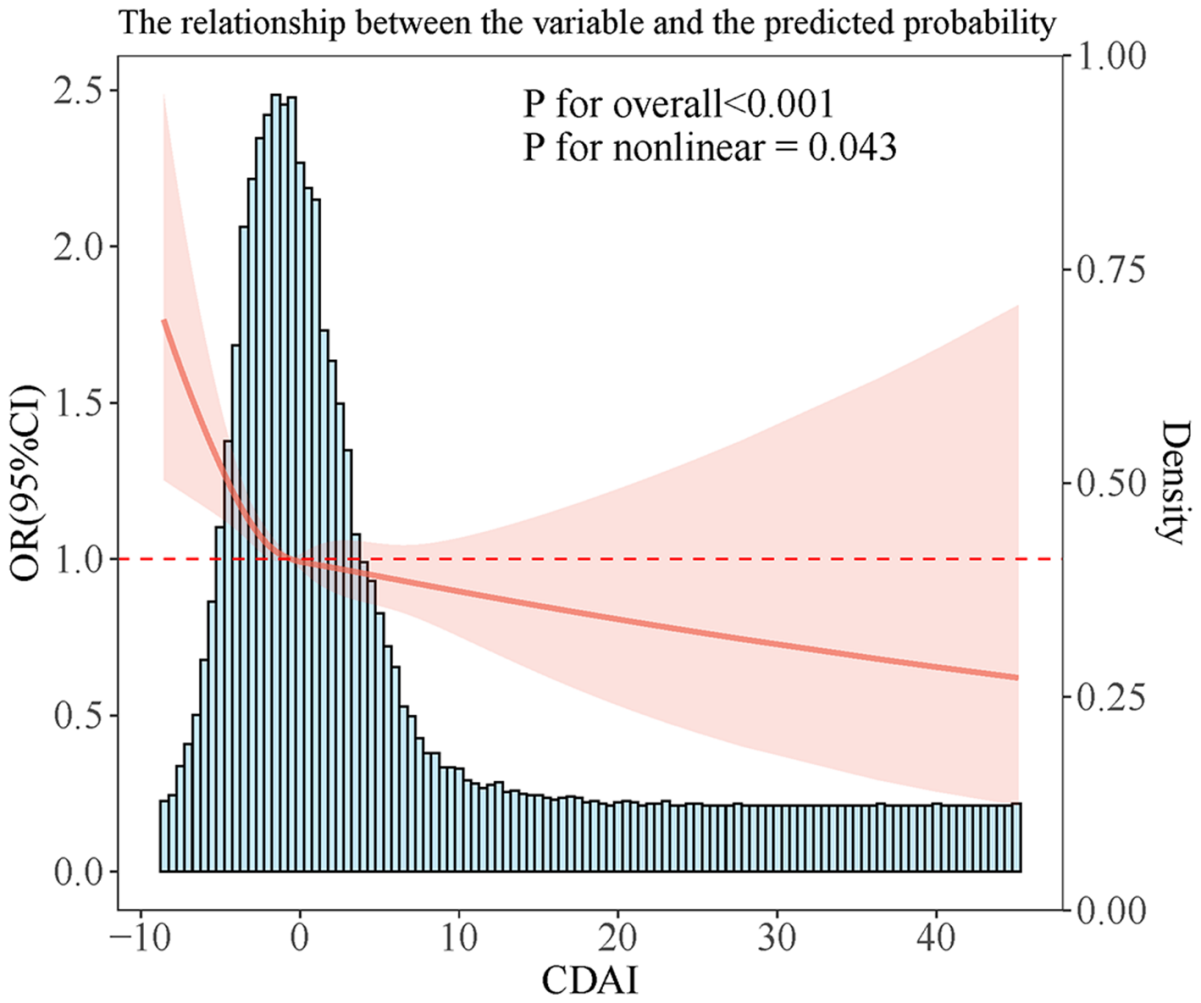
The nonlinear dose–response relationship between CDAI and the risk of HPV infection.

RCS analysis showed a non-linear link (P-non-linear = 0.043). The link showed an L shape, with a CDAI inflection point of −0.57 (Figure 2).

### 3.3 Association between different components of CDAI and HPV infection

The links between different components of CDAI and HPV infection are summarized in Table 3. The associations of Se and Carotenoids with HPV infection were statistically significant only in Model 1 (*p* < 0.05). VA and VC maintained statistical significance in Model 1 and Model 2 (*p* < 0.05). After further adjustment for confounders, Model 3 showed that the associations of VA and VC with HPV infection disappeared (both *p* > 0.05). Notably, only VE and zinc were statistically significant in all 3 models (all *p* < 0.05), and both were negatively associated with HPV infection.

**Table 3:** The relationship between the components of CDAI and HPV.

Participants	Model 1	*p*-value	Model 2	*p*-value	Model 3	*p*-value
	OR (95%CI)		OR (95%CI)		OR (95%CI)	
VA	0.87 (0.79, 0.95)	0.002	0.91 (0.83, 0.98)	0.018	0.97 (0.89, 1.05)	0.400
VC	0.89 (0.83, 0.96)	0.005	0.88 (0.81, 0.95)	0.001	0.92 (0.85, 1.00)	0.057
VE	0.87 (0.81, 0.94)	<0.001	0.90 (0.83, 0.96)	0.004	0.92 (0.86, 0.99)	0.044
Se	0.94 (0.88, 1.01)	0.100	0.94 (0.87, 1.01)	0.081	0.98 (0.91, 1.06)	0.600
Zn	0.86 (0.79, 0.93)	<0.001	0.88 (0.81, 0.95)	0.001	0.92 (0.85, 0.99)	0.038
Carotenoids	0.91 (0.84, 0.98)	0.013	0.93 (0.86, 1.01)	0.077	0.97 (0.90, 1.03)	0.300

No factors were adjusted in Model 1. Model 2 was adjusted for age and race; and Model 3 further considered education level, BMI, marital status, PIR, alcohol consumption, smoking status, DM, oral contraceptives, age at first sex, and male sexual partners in last year. CDAI, comprehensive dietary antioxidant index; HPV, human papillomavirus; OR, odds ratio; CI, confidence interval.

The incidence of each HPV subtype is shown in Appendix Table 1. We further analyzed the effects of VE and zinc intake on HPV subtypes with an incidence ≥1%. The results showed that as zinc intake increased, the risk of HPV-89, 51, 59, 45, and 68 infection decreased, and as VE intake increased, the risk of HPV-66 and 59 infection decreased (Appendix Table 2).

### 3.4 Subgroup analysis

CDAI was inversely linked to HPV infection in groups with BMI ≥ 30 kg/cm^2^, non-Hispanic Black, PIR of 1.3–3, ≥ high school education, married/cohabitation, without DM, and not taking oral contraceptives (*p* < 0.05). We also noted a considerable interaction of marital status in the link (P for interaction = 0.011) (Table 4).

**Table 4:** Subgroup analysis.

Subgroup	OR (95%CI)	*p*-value	p for interaction
BMI, kg/m^2^			0.162
<25	0.99 (0.96, 1.03)	0.731	
25–30	0.99 (0.96, 1.03)	0.509	
≥30	0.96 (0.93, 0.99)	0.011	
Race			0.703
Mexican American	0.97 (0.92, 1.02)	0.199	
Non-Hispanic Black	0.97 (0.94, 0.99)	0.020	
Non-Hispanic White	0.98 (0.95, 1.01)	0.159	
Other Hispanic	0.99 (0.96, 1.03)	0.700	
Other Race	1.01 (0.96, 1.06)	0.610	
PIR			0.309
<1.3	0.98 (0.95, 1.00)	0.077	
1.3–3	0.97 (0.94, 0.99)	0.031	
> = 3	0.99 (0.96, 1.03)	0.621	
Education level			0.376
<High school diploma	1.00 (0.96, 1.04)	0.979	
≥High school diploma	0.98 (0.96, 0.99)	0.032	
Marital status			0.011
Married/cohabitation	0.97 (0.94, 0.99)	0.009	
Unmarried	1.01 (0.98, 1.05)	0.475	
Widow/divorce/separation	0.99 (0.96, 1.03)	0.656	
Alcohol drinking			0.502
Yes	0.98 (0.96, 1.01)	0.177	
No	0.97 (0.93, 1.01)	0.104	
Smoking status			0.733
Current smoker	0.98 (0.95, 1.01)	0.254	
Former smoker	0.99 (0.96, 1.04)	0.796	
Never smoker	0.98 (0.95, 1.00)	0.092	
Diabetes mellitus			0.188
Yes	1.00 (0.95, 1.06)	0.883	
No	0.98 (0.96, 0.99)	0.039	
Have taken birth control pills			0.108
Yes	0.99 (0.97, 1.01)	0.176	
No	0.96 (0.92, 0.99)	0.018	
How old when first had sex, years			0.686
<18	0.98 (0.96, 1.00)	0.050	
≥18	0.98 (0.95, 1.01)	0.280	

Model was adjusted for age, race, education level, BMI, marital status, PIR, alcohol consumption, smoking status, DM, oral contraceptives, age at first sex, and male sexual partners in last year. OR, odds ratio; CI, confidence interval; BMI, body mass index; PIR, poverty-to-income ratio.

### 3.5 Sensitivity analysis

In the sensitivity analysis, Model 3 results showed that for each 1-unit increase in CDAI, the risk of HPV infection decreased by 2.0% [(95% CI: 0.96, 0.99), *p* = 0.024]. We further categorized CDAI into three tertiles. In Models 1 and 2, the T2 and T3 groups showed statistically significant differences compared to the T1 group (*p* < 0.05), with a decreasing trend (P for trend < 0.0001). In Model 3, only the T3 group showed a significant difference compared to the T1 group (*p* = 0.002), also exhibiting a decreasing trend (P for trend = 0.007) (Appendix Table 3).

## 4 Discussion

This paper first evaluated the link between CDAI and HPV infection risk in US women based on the NHANES database. The results showed an inverse link between CDAI and HPV infection risk with a nonlinear dose–response relationship. VE and zinc played protective roles in this process. In addition, marital status had a significant interaction effect on this link.

The present study found a notable interaction between marital/cohabitation status on the link between CDAI and HPV infection risk. Similarly, Jin et al. (22) found that folic acid intake in married or cohabiting populations showed a more pronounced negative link to HPV infection risk. This may stem from the fact that married or cohabiting people usually have more stable dietary habits, higher health awareness, and more regular lifestyles, which enhances the protective effect of dietary nutrition. Our study suggested that marital status or partnership may be an important sociodemographic factor in optimizing dietary antioxidant strategies for the prevention of HPV infection.

Barchitta et al. (16) found that hrHPV infection risk gradually decreased with increasing CDAI, similar to our results. Notably, the present study further revealed a significant nonlinear effect between CDAI and HPV infection. In the lower range of CDAI, HPV infection risk declined greatly with increasing CDAI; however, when CDAI increased above −0.57, the decrease leveled off significantly, although the infection risk continued to decrease. This non-linear trend suggests that the protective effect of dietary antioxidants against HPV infection may gradually become saturated after reaching a certain threshold. This important finding extends and enriches the conclusions by Barchitta et al. (16). It suggests that the appropriate dosage and range of antioxidant supplementation or dietary modification need to be considered in clinical interventions.

The present study found that VE intake was negatively related to HPV infection risk, consistent with the findings of previous studies. A Brazilian cohort study involving 405 women (23) showed that serum VE levels may have a protective effect against persistent non-cancerous HPV infection. Another study based on the NHANES database, involving 5,809 American women (24), further found that VE intake had a protective effect against both lrHPV and hrHPV infection. It is worth noting that despite significant differences in dietary patterns between Brazil and the United States—for example, Brazilian diets tend to include higher intakes of tropical fruits, nuts, and vegetable oils, while American diets tend to include higher proportions of processed foods and high-fat animal-based foods—both studies consistently observed a negative correlation between VE intake and HPV infection risk. This suggests that regardless of the dietary source, as long as the VE intake reaches a certain level, it may have a protective effect against persistent HPV infection. VE can directly scavenge ROS and RNS, block the free radical chain reaction of lipid peroxidation, and thus protect the polyunsaturated fatty acids in cell membranes from oxidative damage. At the same time, VE can activate the Nrf2/ARE signaling pathway to upregulate the expression of multiple antioxidant defense enzymes, thereby enhancing the overall antioxidant capacity of cells. In addition, VE and its metabolites can further improve the reducing state of cells by increasing levels of glutathione and antioxidant-related transcription factors. Through these direct and indirect mechanisms, VE can effectively alleviate oxidative stress, reduce DNA oxidative damage, and even play a potential inhibitory role in tumorigenesis (25). In addition, we unveiled a negative link between zinc intake and HPV infection, consistent with the findings of Xiao et al. (26). Zinc plays a key role in immune regulation, antioxidant activity, and inhibition of viral replication in the body (27, 28). Zinc can directly regulate the activity of immune cells (such as DCs, NK cells, and T cells) and enhance antiviral immune responses by regulating signaling pathways such as NF-κB and PKC/Lck, thereby promoting viral clearance (29). Furthermore, zinc can exert antioxidant effects through multiple pathways (30). Research shows that zinc can downregulate the expression of HPV oncogenic proteins E6/E7 and induce apoptosis in cancerous cells by activating the p53 signaling pathway (31, 32).

Previous research has stated the protective role of VA, VC, Se, and carotenoid intake in HPV infection, cervical dysplasia, and cervical cancer progression (9, 33, 34). However, no negative correlation was revealed in our study, and this discrepancy may be due to multiple factors. For example, Huang et al. (11) highlighted a U-shaped link between VA intake and HPV infection. When VA intake was lower than 1448.155 mcg, there was a negative link to HPV infection, and a positive link was found when VA intake exceeded this threshold. The VA intake of the participants in this paper was 506.50 mcg (IQR:317.50–764.50), but there was a subset of individuals whose VA intake exceeded this threshold. Zheng et al. (10) elicited that serum VC was inversely linked to HPV infection only in females ≥25 years. In addition, these factors may have attenuated the overall link between antioxidants and HPV infection, given the interaction of dietary antioxidants.

This paper has several strengths. First, it was based on the U.S. NHANES database, with a large sample size (8,115 women included), national representativeness, and high generalizability of the findings. Second, this study used the CDAI, a composite indicator, which can reflect the overall level of dietary antioxidant intake more comprehensively compared with single nutrients. In addition, multivariate weighted logistic regression was leveraged to fully consider possible confounders, and further subgroup analyzes were performed. Moreover, the nonlinear dose–response link between CDAI and HPV infection was analyzed via the RCS method, which helped to clarify the specific dose effect and provided important references for the application of dietary antioxidants in the prevention and control of HPV infection.

However, this study also has certain shortcomings. First, similar to the Barchitta study (16), the present study was limited by a cross-sectional design, which restrains a clear causal inference. Second, the data on dietary antioxidant intake were sourced from 24 h recalls, which may have recall biases and measurement errors. In addition, despite adjusting for multiple covariates in the multivariate model, there may still be residual confounders that were not measured or included in the model, such as genetic factors and duration of HPV exposure. Further prospective cohort articles or RCTs are needed to offer a more reliable basis for the precision prevention of HPV infection.

## 5 Conclusion

CDAI is significantly negatively linked to HPV infection risk, with an L-shaped nonlinear dose–response link. It is recommended that U.S. women increase their intake of antioxidant-rich diets, especially foods rich in zinc and VE, to reduce HPV infection risk.

## Data Availability

The raw data supporting the conclusions of this article will be made available by the authors, without undue reservation.
