# Supplementary material for: An L-shaped link between the composite dietary antioxidant index and human papillomavirus infection in women: a US population-based study
Source: Front Nutr. 2025 Sep 10;12:1604908. doi: 10.3389/fnut.2025.1604908 (PMC12457662; doi:10.3389/fnut.2025.1604908)
Supplement: Supplementary file 1 [file Table_1.DOCX]

**Appendix Table 1** HPV Infection Status by Type

| **Genotype** | **Positive n** | **Prevalence percent** | **Included** |
| --- | --- | --- | --- |
| HPV type 62 | 470 | 5.79 | Yes |
| HPV type 53 | 458 | 5.64 | Yes |
| HPV type 61 | 443 | 5.46 | Yes |
| HPV type 54 | 378 | 4.66 | Yes |
| HPV type 84 | 371 | 4.57 | Yes |
| HPV type 89 | 369 | 4.55 | Yes |
| HPV type 83 | 323 | 3.98 | Yes |
| HPV type 16 | 317 | 3.91 | Yes |
| HPV type 52 | 263 | 3.24 | Yes |
| HPV type 51 | 261 | 3.22 | Yes |
| HPV type 66 | 224 | 2.76 | Yes |
| HPV type 81 | 222 | 2.74 | Yes |
| HPV type 70 | 205 | 2.53 | Yes |
| HPV type 59 | 200 | 2.46 | Yes |
| HPV type 39 | 192 | 2.37 | Yes |
| HPV type 55 | 189 | 2.33 | Yes |
| HPV type 42 | 169 | 2.08 | Yes |
| HPV type 56 | 162 | 2 | Yes |
| HPV type 45 | 153 | 1.89 | Yes |
| HPV type 73 | 151 | 1.86 | Yes |
| HPV type 31 | 149 | 1.84 | Yes |
| HPV type 35 | 148 | 1.82 | Yes |
| HPV type 58 | 147 | 1.81 | Yes |
| HPV type 68 | 147 | 1.81 | Yes |
| HPV type 18 | 143 | 1.76 | Yes |
| HPV type 72 | 134 | 1.65 | Yes |
| HPV type 06 | 132 | 1.63 | Yes |
| HPV type 71 | 72 | 0.89 | No |
| HPV type 67 | 67 | 0.83 | No |
| HPV type 40 | 61 | 0.75 | No |
| HPV type 33 | 54 | 0.67 | No |
| HPV type 82 | 38 | 0.47 | No |
| HPV type 69 | 26 | 0.32 | No |
| HPV type IS39 | 23 | 0.28 | No |
| HPV type 26 | 18 | 0.22 | No |
| HPV type 11 | 14 | 0.17 | No |
| HPV type 64 | 7 | 0.09 | No |

"Positive n" refers to a positive event.

HPV: human papillomavirus

**Appendix Table 2** Relationship between zinc/VE intake and different HPV subtypes

| **Participants** | **Model** |  |
| --- | --- | --- |
|  | **OR (95%CI)** | ***p*-value** |
| **Zn** |  |  |
| HPV type 62 | 1.07(0.93, 1.22) | 0.346 |
| HPV type 53 | 0.88(0.76, 1.03) | 0.108 |
| HPV type 61 | 0.98(0.82, 1.16) | 0.779 |
| HPV type 54 | 0.87(0.72, 1.04) | 0.115 |
| HPV type 84 | 0.90(0.78, 1.04) | 0.142 |
| HPV type 89 | 0.79(0.66, 0.94) | 0.008 |
| HPV type 83 | 0.83(0.68, 1.02) | 0.080 |
| HPV type 16 | 0.99(0.83, 1.17) | 0.878 |
| HPV type 52 | 1.04(0.89, 1.20) | 0.640 |
| HPV type 51 | 0.80(0.67, 0.95) | 0.022 |
| HPV type 66 | 0.89(0.70, 1.13) | 0.332 |
| HPV type 81 | 0.94(0.78, 1.12) | 0.478 |
| HPV type 70 | 0.91(0.70, 1.18) | 0.451 |
| HPV type 59 | 0.72(0.58, 0.90) | 0.004 |
| HPV type 39 | 1.04(0.84, 1.30) | 0.699 |
| HPV type 55 | 0.98(0.74, 1.30) | 0.875 |
| HPV type 42 | 0.94(0.76, 1.17) | 0.568 |
| HPV type 56 | 0.89(0.71, 1.11) | 0.287 |
| HPV type 45 | 0.74(0.54, 0.99) | 0.049 |
| HPV type 73 | 0.83(0.64, 1.07) | 0.157 |
| HPV type 31 | 0.83(0.59, 1.16) | 0.261 |
| HPV type 35 | 0.87(0.65, 1.16) | 0.334 |
| HPV type 58 | 1.16(0.93, 1.46) | 0.189 |
| HPV type 68 | 0.74(0.59, 0.94) | 0.013 |
| HPV type 18 | 1.01(0.78, 1.30) | 0.965 |
| HPV type 72 | 0.82(0.58, 1.16) | 0.269 |
| HPV type 06 | 0.76(0.57, 1.02) | 0.071 |
| **VE** |  |  |
| HPV type 62 | 1.00(0.87, 1.16) | 0.955 |
| HPV type 53 | 0.92(0.81, 1.04) | 0.203 |
| HPV type 61 | 0.91(0.79, 1.04) | 0.155 |
| HPV type 54 | 0.96(0.82, 1.12) | 0.563 |
| HPV type 84 | 0.85(0.70, 1.03) | 0.101 |
| HPV type 89 | 1.04(0.87, 1.24) | 0.684 |
| HPV type 83 | 0.96(0.83, 1.13) | 0.650 |
| HPV type 16 | 0.96(0.77, 1.20) | 0.735 |
| HPV type 52 | 0.99(0.86, 1.15) | 0.930 |
| HPV type 51 | 0.87(0.70, 1.08) | 0.210 |
| HPV type 66 | 0.76(0.60, 0.97) | 0.025 |
| HPV type 81 | 0.89(0.75, 1.06) | 0.202 |
| HPV type 70 | 0.96(0.81, 1.14) | 0.674 |
| HPV type 59 | 0.71(0.55, 0.92) | 0.011 |
| HPV type 39 | 0.89(0.68, 1.15) | 0.369 |
| HPV type 55 | 0.98(0.78, 1.20) | 0.822 |
| HPV type 42 | 0.95(0.74, 1.22) | 0.704 |
| HPV type 56 | 1.05(0.91, 1.21) | 0.474 |
| HPV type 45 | 0.81(0.63, 1.04) | 0.094 |
| HPV type 73 | 0.91(0.71, 1.17) | 0.464 |
| HPV type 31 | 0.92(0.72, 1.19) | 0.532 |
| HPV type 35 | 0.90(0.67, 1.18) | 0.425 |
| HPV type 58 | 1.11(0.88, 1.39) | 0.381 |
| HPV type 68 | 1.04(0.86, 1.27) | 0.677 |
| HPV type 18 | 1.08(0.92, 1.27) | 0.318 |
| HPV type 72 | 1.08(0.89, 1.32) | 0.410 |
| HPV type 06 | 1.01(0.82, 1.24) | 0.927 |

Model was adjusted for age, race, education level, BMI, marital status, PIR, alcohol consumption, smoking status, DM, oral contraceptives, age at first sex, and male sexual partners in last year.

HPV: human papillomavirus, Zn: zinc, VE: vitamin E, OR: odds ratio, CI: confidence interval

**Appendix Table 3** The relationship between CDAI and HPV. (Excluding those who have received the HPV vaccine)

| **Participants** | **Model 1** |  | **Model 2** |  | **Model 3** |  |
| --- | --- | --- | --- | --- | --- | --- |
|  | **OR (95%CI)** | ***p*-value** | **OR (95%CI)** | ***p*-value** | **OR (95%CI)** | ***p*-value** |
| CDAI | 0.95(0.94, 0.97) | ＜0.001 | 0.97(0.95, 0.99) | 0.002 | 0.98(0.96, 0.99) | 0.024 |
| Tertiles |  |  |  |  |  |  |
| T1 | Ref |  | Ref |  | Ref |  |
| T2 | 0.72(0.60, 0.86) | ＜0.001 | 0.82(0.68,0.98) | 0.032 | 0.88(0.73, 1.05) | 0.158 |
| T3 | 0.61(0.52, 0.72) | ＜0.001 | 0.71(0.60, 0.84) | ＜0.001 | 0.76(0.63, 0.90) | 0.002 |
| *p* for trend | ＜0.001 |  | ＜0.001 |  | 0.007 |  |

No factors were adjusted in Model 1. Model 2 was adjusted for age and race; and Model 3 further considered education level, BMI, marital status, PIR, alcohol consumption, smoking status, DM, oral contraceptives, age at first sex, and male sexual partners in last year.

CDAI: comprehensive dietary antioxidant index, HPV: human papillomavirus, OR: odds ratio, CI: confidence interval
